# Functional consequences of TCF4 missense substitutions associated with Pitt-Hopkins syndrome, mild intellectual disability, and schizophrenia

**DOI:** 10.1016/j.jbc.2021.101381

**Published:** 2021-11-06

**Authors:** Alex Sirp, Kaisa Roots, Kaja Nurm, Jürgen Tuvikene, Mari Sepp, Tõnis Timmusk

**Affiliations:** 1 Department of Chemistry and Biotechnology, Tallinn University of Technology, Tallinn, Estonia; 2 Protobios LLC, Tallinn, Estonia

**Keywords:** neurocognitive disorders, schizophrenia, intellectual disability, Pitt-Hopkins syndrome, transcription factor TCF4, missense mutation, single-nucleotide polymorphism, autism, basic helix-loop-helix transcription factor, neuron, AD, activation domain, ASCL1, achaete-scute homolog 1, bHLH, basic helix-loop-helix, BSA, bovine serum albumin, CE, conserved element, MMID, Mild-to-moderate intellectual disability, NLS, Nuclear localization signal, PBST, PBS with Tween 20, PGK, phosphoglycerine kinase, PTHS, Pitt-Hopkins syndrome, RTT-like, Rett-like syndrome, SCZ, Schizophrenia, TCF4, Transcription factor 4, TK, thymidine kinase

## Abstract

Transcription factor 4 (TCF4) is a basic helix-loop-helix transcription factor essential for neurocognitive development. The aberrations in *TCF4* are associated with neurodevelopmental disorders including schizophrenia, intellectual disability, and Pitt-Hopkins syndrome, an autism-spectrum disorder characterized by developmental delay. Several disease-associated missense mutations in *TCF4* have been shown to interfere with TCF4 function, but for many mutations, the impact remains undefined. Here, we tested the effects of 12 functionally uncharacterized disease-associated missense mutations and variations in *TCF4* using transient expression in mammalian cells, confocal imaging, *in vitro* DNA-binding assays, and reporter assays. We show that Pitt-Hopkins syndrome-associated missense mutations within the basic helix-loop-helix domain of TCF4 and a Rett-like syndrome-associated mutation in a transcription activation domain result in altered DNA-binding and transcriptional activity of the protein. Some of the missense variations found in schizophrenia patients slightly increase TCF4 transcriptional activity, whereas no effects were detected for missense mutations linked to mild intellectual disability. We in addition find that the outcomes of several disease-related mutations are affected by cell type, TCF4 isoform, and dimerization partner, suggesting that the effects of TCF4 mutations are context-dependent. Together with previous work, this study provides a basis for the interpretation of the functional consequences of *TCF4* missense variants.

Transcription factor 4 (TCF4) is vital for normal development of the central nervous system, and the mutations within the *TCF4* gene have been linked to several neurodevelopmental diseases such as Pitt-Hopkins syndrome (PTHS), mild-to-moderate intellectual disability (MMID), and schizophrenia (SCZ) (1). A single missense mutation in the basic helix-loop-helix (bHLH) region, which mediates dimerization and DNA binding, is enough to cause PTHS: a rare syndromic encephalopathy characterized by severe intellectual disability, autistic-like behaviour, distinct facial features, breathing abnormalities, absent language, motor deficits, and epilepsy (2, 3, 4). In addition, a variety of *TCF4 de novo* translocations, deletions, insertions, nonsense, frame-shift, and splice-site mutations which affect overall *TCF4* expression or the functionality of the bHLH domain have been identified in PTHS patients (5, 6, 7). *De novo* partial deletions, translocations, missense, and truncating mutations in *TCF4* have also been identified in MMID patients without the typical characteristics of PTHS (8, 9, 10, 11, 12, 13) and one missense mutation in TCF4 has been linked to a Rett-like syndrome (RTT-like) (14). *TCF4* is one of the susceptibility genes for SCZ with several intronic SNPs (15, 16, 17, 18, 19) and the missense variations (20, 21) in *TCF4* found to be associated with the disease. In addition to neurodevelopmental diseases, *TCF4* is one of the most frequently mutated genes in adult sonic-hedgehog-driven medulloblastoma, a tumor of the cerebellum (22, 23).

As a class I bHLH transcription factor, TCF4 forms homo or heterodimers with other bHLH transcription factor family members and binds to the Ephrussi box consensus sequence (CANNTG) (24, 25, 26). The overall regulation of TCF4 activity is complex and relies on the expression pattern of its activating and inhibiting interaction partners throughout development (27, 28). The differential expression of interaction partners allows TCF4 to exert various functions necessary for normal brain development such as apoptosis, proliferation, signaling, and migration (29, 30, 31). For example, TCF4 heterodimerizes with achaete-scute homolog 1(ASCL1) (32) and regulates the differentiation of neural stem cells (33).

Over 18 different protein isoforms are encoded by the human *TCF4* gene (34). The most studied TCF4 isoforms to date are TCF4-B and TCF4-A, which represent a long and a short TCF4 isoform, respectively (Fig. 1*A*). All TCF4 isoforms exhibit the evolutionally conserved bHLH domain and activation domains 2 and 3 (AD2 and AD3), but only long isoforms have an additional activation domain 1 (AD1) (34, 35). As a result, the longer isoform TCF4-B, that exhibits full-length AD1, has a higher transcriptional activity than the shorter isoform TCF4-A, that lacks AD1 (34, 36). The TCF4 activation domains are regulated by the so-called conserved element (CE), which can repress AD1-dependent transcription (37), and the transcriptional repression domain which can repress both AD1 and AD2 (38). Motifs A and C on either side of the bHLH domain regulate homo and heterodimerization (39, 40). The nuclear localization of TCF4 is mediated by two nuclear localization signals (NLS): a bipartite NLS-1 coded by exons 8 and 9 (34), which can also function as nucleolar localization signal, and a recently identified NLS-2 inside the bHLH domain (41).

**Figure 1:**
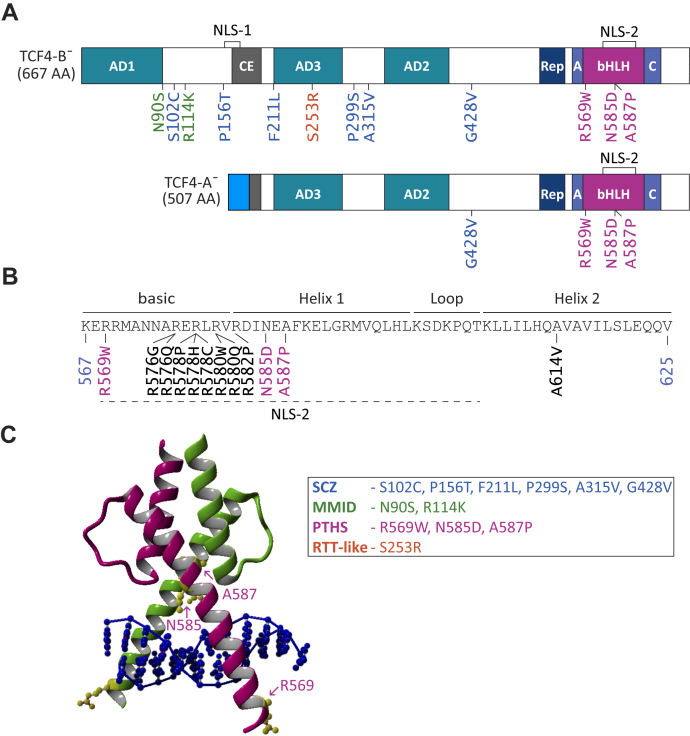
**Localization of disease-related missense variations and mutations in TCF4.***A*, representation of TCF4 isoforms B¯ and A¯. The *colored boxes* show conserved protein domains. The *dark-grey* region in TCF4-A¯ represents a nonfunctional partial CE domain (according to Herbst and Kolligs (37)) and the *blue region* in TCF4-A¯ shows unique amino acids encoded by exon 10a of *TCF4* (34). Nuclear localization signals are shown on *top*. Disease-related missense substitutions analyzed in the present article are shown below the protein isoforms and marked according to the color code below. Missense mutations and variations have been associated with mild-to-moderate intellectual disability (MMID; N90S and R114K, marked *green*), schizophrenia (SCZ; S102C, P156T, F211L, P299S, A315V, and G428V, marked *blue*), Rett-like syndrome (RTT-like; S253R, marked *orange*), and Pitt-Hopkins syndrome (PTHS; R569W, N585D, and A587P, marked *purple*). *B*, the position of missense mutations in TCF4 bHLH identified in PTHS patients to date. Novel PTHS-associated missense mutations are shown in *purple*. The protein regions are indicated with *lines* on *top*, and NLS-2 is marked with a *dashed underline*. The bHLH beginning (567) and end (625) coordinates are in the context of TCF4-B^+^. *C*, ribbon drawing of TCF4 structure model (5) showing bHLH homodimer bound to DNA (*dark blue*). *Pink* and *green* structures indicate the ‘nonspecific’ and ‘specific’ TCF4 subunit, respectively. The novel missense substitutions identified in PTHS patients are colored *yellow* and indicated with *arrows*. A, motif A; bHLH, basic helix-loop-helix; C, motif C; CE, conserved element; AD1, AD2, and AD3, transcription activation domain; NLS, nuclear localization signal; Rep, repression domain; TCF4, transcription factor 4.

TCF4 is broadly expressed, but its mRNA levels vary in different tissues with high expression detected in different brain regions (34, 42, 43, 44, 45). Homozygous deletion of *Tcf4* is lethal in mice (46). *Tcf4* haplo-insufficient mice display impaired learning and motor control, memory deficits, social isolation (47) and microcephaly, hyperactivity, reduced anxiety, and enhanced long-term potentiation (48, 49). *In utero TCF4* gain-of-function studies in rats lead to enhanced spontaneous activity of prefrontal neurons and disruption of the formation of prefrontal cortical minicolumns (44). *In utero* suppression of *Tcf4* in rats leads to decreased excitability of prefrontal neurons (50). Overexpression of TCF4 in the mouse brain causes impairments in cognition and sensorimotor gating (51) and enhanced long-term depression (48). In addition, the overexpression of Daughterless, the *Drosophila melanogaster* orthologue of TCF4, is lethal in adult flies, whereas the downregulation of Daughterless impairs memory and learning (52, 53).

It is known that PTHS is caused by loss-of-function mutations leading to *TCF4* haploinsufficiency (7). It is less known how TCF4 may be involved in the development of MMID and SCZ, however, evidence suggests that it may be because of changes in TCF4 dosage. MMID is caused by mutations located in the 5′ region of the *TCF4* gene thus only affecting the expression of long TCF4 isoforms (12). In the case of SCZ, the studies suggest that *TCF4* expression levels are elevated ((54) medRxiv, (55)). Functional impact of many of the PTHS-associated missense mutations have been studied before (4, 5, 45, 56). According to these studies, mutant TCF4 proteins display changes in DNA binding, dimerization, transcription activation, and intranuclear localization. The effects range from hypomorphic to dominant negative, and the missense mutations located in the bHLH domain have the most severe functional impact. In addition, the variations associated with SCZ (located outside the bHLH region) have been shown to increase the activity of TCF4 (36).

Several novel disease-related missense variations and the mutations associated with SCZ, MMID, RTT-like syndrome, and PTHS have been identified in TCF4, however, the functional impact of these substitutions has not been studied. Here, we investigated the effects of previously uncharacterized disease-related missense mutations in TCF4 on its dimerization, DNA binding, and transactivation ability and on its subcellular distribution. Our results extend current knowledge on how amino acid substitutions in TCF4 can affect the functionality of the protein.

## Results

### Mapping of disease-related missense mutations in TCF4

Several missense variations and mutations across the *TCF4* coding sequence have been identified in MMID, SCZ, RTT-like syndrome, and PTHS patients. For the present study, we selected 12 functionally undescribed missense variations and mutations, numbered them in the context of the full-length isoform TCF4-B^+^, and mapped them onto isoforms TCF4-B¯ and TCF4-A¯ (Fig. 1*A*). Schizophrenia-associated variations have been described in two separate studies (20, 21). All but one of the schizophrenia-associated missense variations have been identified in a single patient and no controls, however, the variation A315V has been found in 14 patients and 10 controls. The variations S102C, F211L, P299S, A315V, and G428V are located in exons 6, 10, 12, 13, and 16, respectively. These variations do not map to any known functionally important region of TCF4 protein. The variation P156T is in exon 8 in front of NLS-1 coding sequence. MMID mutations N90S and R114K (Pitt-Hopkins Research Foundation, Audrey Davidow Lapidus, personal communication) are in *TCF4* exons 6 and 7, respectively. N90S is located at the end of AD1, whereas R114K is not located to any functionally important domain of TCF4. The RTT-like syndrome mutation S253R (14) is in exon 11 in AD3 coding sequence (Fig. 1*A*).

Missense mutations in PTHS patients are predominantly, but not always, located in the bHLH domain of TCF4. Previous studies have identified 12 amino acid substitutions in eight positions of the TCF4 bHLH region in 25 PTHS patients (Table 1, Fig. 1*B*). Most of the amino acid substitutions affect four arginines (R569, R576, R578, and R580) in the basic region of the bHLH domain. Three missense mutations (R582P, N585D, and A587P) are in helix 1 and one missense mutation (A614V) in helix 2. Of these, we have previously functionally characterized mutations at R576, R578, and R580 in the basic region, R582 in helix 1, and A614 in helix 2. These mutations showed varied severity ranging from hypomorphic effects to complete loss-of-function (5). Here, we addressed the functional impact of three additional amino acid substitutions R569W, N585D, and A587P (6, 57). We began by estimating possible effects of these missense mutations based on previously generated bHLH structure model (5). R569 resides in the beginning of the basic region and according to the model, contacts DNA at an E-box flanking region. N585 and A587 are both located in helix 1 of the bHLH domain. In the TCF4 model, the amino acid N585 forms hydrogen bonds with the DNA backbone whereas A587 packs against residue L611 in helix 2 of the dimerization partner (Fig. 1*C*).

**Table 1:** List of missense mutations in TCF4 basic helix-loop-helix domain identified in Pitt-Hopkins syndrome patients to date

No	*TCF4* mutations	AA changes	References
1	1705 C>T	R569W	Whalen *et al.*, 2012 (6)
2	1726 C>G	R576G	de Pontual *et al.*, 2009 (45)
3	1727 G>A	R576Q	de Pontual *et al.*, 2009 (45)
4	1732 C>T	R578C	Marangi *et al.*, 2012 (57)
5	1733 G>C	R578P	Zweier *et al.*, 2008 (67)
6	1733 G>A	R578H	Zweier *et al.*, 2008 (67)
7	1738 C>T	R580W	Amiel *et al.*, 2007 (2); Zweier *et al.*, 2007 (4)
8	1739 G>A	R580Q	Amiel *et al.*, 2007 (2)
9	1745 G>C	R582P	Takano *et al.*, 2010 (68)
10	1753 A>G	N585D	Marangi *et al.*, 2012 (57)
11	1759 G>C	A587P	Whalen *et al.*, 2012 (6)
12	1841 C>T	A614V	de Pontual *et al.*, 2009 (45)

Only one SCZ (P156T), MMID (N90S), and RTT-like syndrome (S253R) related missense mutation is in a functionally important domain of TCF4. However, all PTHS-associated missense mutations in the bHLH domain affect conserved amino acids (5, 52), suggesting a high importance of those residues in modulating dimerization, DNA binding, and/or nuclear localization.

### Intranuclear localization of TCF4-B¯ is altered by PTHS-associated missense mutations

**Figure 2:**
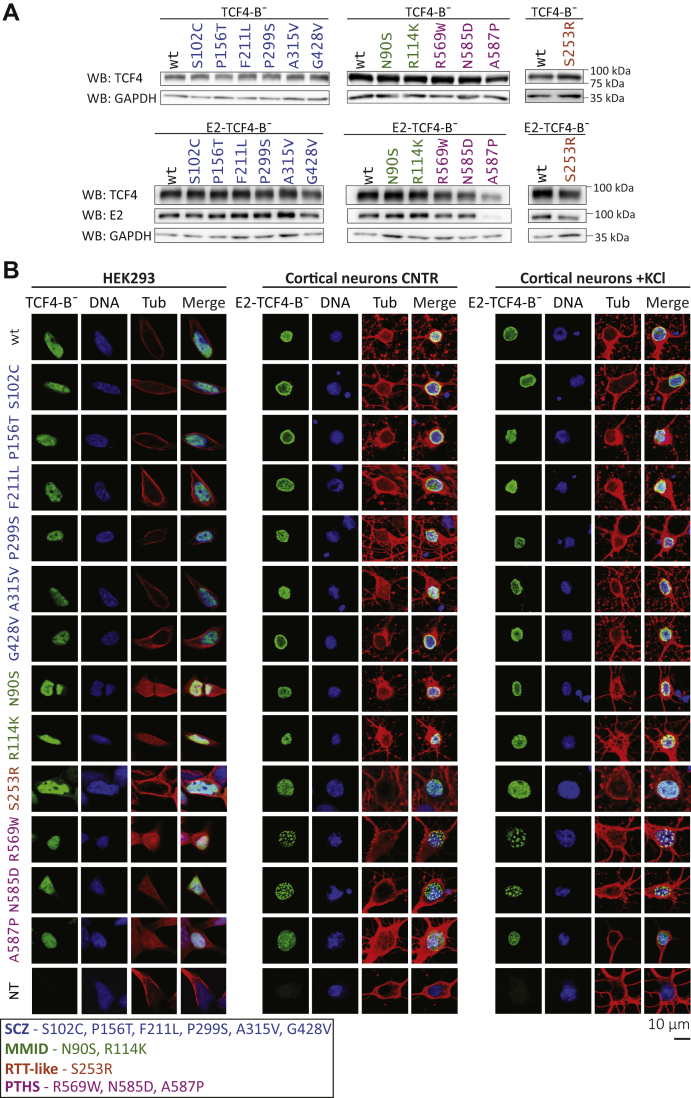
**Intracellular localization of WT and mutant TCF4-B¯ in cultured cells.***A*, Western blot analysis of HEK293 cells transfected with untagged or E2-tagged WT or mutant TCF4-B¯. TCF4 and GAPDH (loading control) signals were detected with specific antibodies. Molecular mass markers are indicated on the *right*. *B*, immunocytochemical analysis of WT or mutant TCF4-B¯ overexpressed in HEK293 cells and of WT or mutant E2-TCF4-B¯ overexpressed in rat hippocampal and cortical primary neurons under basal (CNTR) and depolarized (+KCl) conditions. TCF4, nuclei, and cytoskeleton were visualized with anti-TCF4 or anti-E2 antibody (*green*, indicated as TCF4-B¯), Hoechst 33342 (*blue*, indicated as DNA), and anti-tubulin-β antibody (*red*, indicated as Tub), respectively. The lack of signal in cells not transfected with TCF4 constructs (NT) demonstrates the specificity of anti-TCF4 (HEK293 cells) and anti-E2 (cortical neurons) antibodies. The representative confocal microscopy images are shown. HEK, human embryonic kidney; MMID, Mild-to-moderate intellectual disability; PTHS, Pitt-Hopkins syndrome; RTT-like, Rett-like syndrome; SCZ, Schizophrenia; TCF4, transcription factor 4.

To dissect the functional impact of the novel disease-related TCF4 missense mutations, we introduced point mutations into the TCF4-B¯ expression vector *via* site-directed mutagenesis and transfected WT and mutant constructs into HEK293 cells. Western blot analysis revealed that both the untagged and E2-tagged mutant proteins were overexpressed at comparable levels in HEK293 cells, although A587P mutant proteins displayed lower expression levels than the WT protein (Fig. 2*A*).

Previous studies have shown that the long TCF4-B isoform contains two NLSs located at the amino acids R157-K175 (NLS-1) and R569-K607 (NLS-2) (34, 41). We hypothesized that schizophrenia-associated missense variation P156T could disrupt the N-terminal bipartite NLS-1, whereas PTHS-associated mutations could have an impact on the function of the NLS-2 located in the bHLH region.

We studied the localization of TCF4 proteins in HEK293 cells and in cultured rat cortical and hippocampal primary neurons transfected with WT or mutant TCF4-B¯ expression constructs. Because TCF4 is highly expressed in the brain (42), we used E2-tagged TCF4-B¯ coding constructs in experiments with primary neurons. The use of E2-tagged proteins avoided the simultaneous detection of overexpressed and endogenous TCF4 in primary neurons, where endogenous TCF4 levels are much higher than in HEK293 cells. As TCF4 is an activity-regulated transcription factor (36), we in addition studied TCF4-B¯ localization in neurons treated with 25 mM KCl for 8 h to induce neuronal activity. Our results show that all mutant TCF4-B¯ proteins localize to the cell nucleus both in HEK293 cells and in primary neurons, similarly to WT TCF4-B¯ (Fig. 2*B*). However, in contrast to the homogenous intranuclear distribution of the WT protein, R569W and N585D mutants were detected in the nuclei of primary neurons as puncta, which were universally present in all transfected cells and remained unchanged in response to KCl treatment (Fig. 2*B*). We conclude that the studied disease-related missense substitutions in TCF4-B¯ do not affect nuclear import of the protein, but the PTHS-associated mutations R569W and N585D modify its distribution inside the nuclei of neurons.

### Heterodimerization capacity of TCF4-A is not impaired by mutations in the C-terminal region of the protein

**Figure 3:**
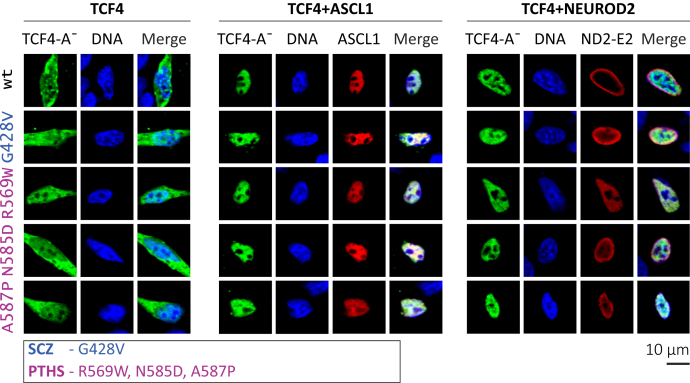
**Heterodimerization of WT or mutant TCF4-A¯ with ASCL1 and NEUROD2 in HEK293 cells.** Nuclear redirection assay with WT or mutant TCF4-A¯ proteins overexpressed in HEK293 cells alone, together with ASCL1 or together with NEUROD2-E2 (ND2-E2). Immunocytochemical staining was carried out with anti-TCF4 (*green*, indicated as TCF4-A¯), anti-MASH1 (*red*, indicated as ASCL1), and anti-E2 (*red*, indicated as ND2-E2) antibodies, and the nuclei were visualized with Hoechst 33342 (*blue*, indicated as DNA). The images were taken by confocal microscopy. HEK, human embryonic kidney; PTHS, Pitt-Hopkins syndrome; SCZ, Schizophrenia; TCF4, transcription factor 4.

We next investigated the possibility that disease-related missense mutations in TCF4 could interfere with the ability of TCF4 to form heterodimers with its well-known dimerization partners such as the class II bHLH transcription factors ASCL1 and NEUROD2, which are involved in the regulation of cell-type-specific transcription (32, 51). To study the heterodimerization capacity of mutant TCF4 proteins, we took advantage of the knowledge that the overexpressed TCF4-A¯ is located both in the cytoplasm and nucleus, and localization strictly to the nucleus is accomplished *via* heterodimerization with NLS-bearing dimerization partners (5). In these experiments, we focused on the four C-terminal mutations located within or close to the bHLH domain. Schizophrenia-related G428V and PTHS-associated R569W, N585D and A587P were introduced into TCF4-A¯ vector (Fig. 1*A*) and expressed alone or together with ASCL1 or NEUROD2-E2 in HEK293 cells. Immunocytochemical analysis demonstrated that similarly to WT TCF4-A¯, all mutant proteins localized both in the cytoplasm and in the nucleus, possibly because of heterodimerization with endogenously expressed dimerization partners or NLS-2 (Fig. 3). The coexpression with ASCL1 or NEUROD2-E2 caused the mutant TCF4-A¯ proteins to localize strictly into the cell nucleus, and no signal was detected in the cytoplasm (Fig. 3). These results show that the four studied disease-related missense substitutions do not disrupt TCF4 heterodimerization.

### PTHS and RTT-like syndrome-associated mutations in TCF4 decrease its DNA-binding activity in a dimerization context-dependent manner

**Figure 4:**
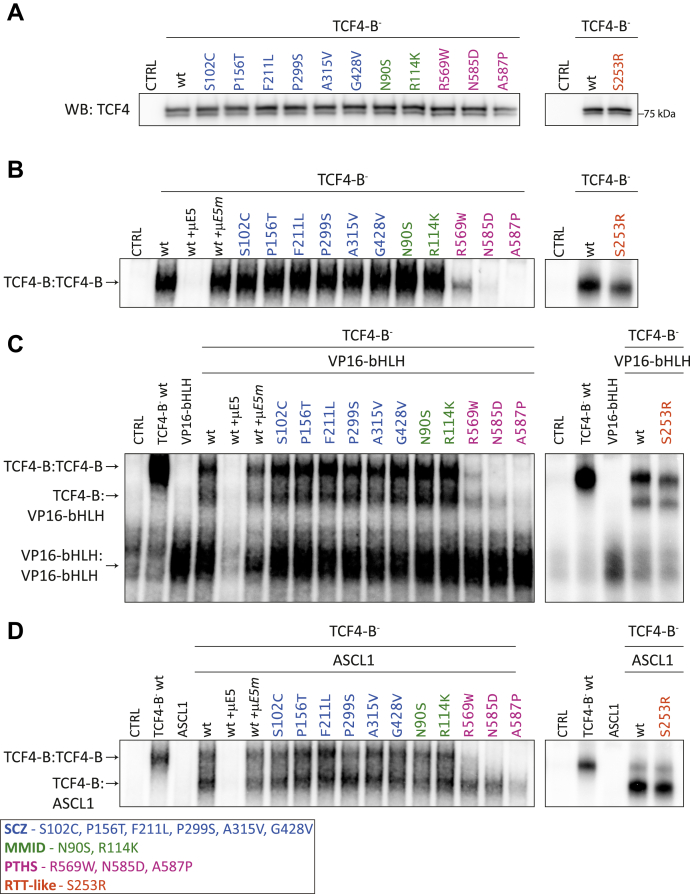
**DNA binding of TCF4 homo and heterodimers is impaired by mutations associated with PTHS.***A*, Western blot analysis of *in vitro*-translated WT or disease-associated missense variations or mutations containing TCF4-B¯ proteins. Molecular mass marker is shown on the *right*. *B*–*D*, EMSA to study the binding of *in vitro*-translated WT or mutant TCF4-B¯ proteins to the 32^P^-labeled μE5 E-box (CACCTG) containing oligonucleotide as (*B*) homodimers (TCF4-B:TCF4-B), (*C*) intra-TCF4 heterodimers consisting of one WT or mutant TCF4-B¯ and one WT VP16-bHLH subunit (TCF4-B:VP16-bHLH), and (*D*) heterodimers with ASCL1 (TCF4-B:ASCL1). The unlabeled WT (μE5) or mutated (μE5m) E-box oligonucleotides were added to the binding mixture for competition where indicated in *italics*. The uncropped EMSA images can be found in Figure S1. bHLH, basic helix-loop-helix; kDa, kilodalton; MMID, Mild-to-moderate intellectual disability; PTHS, Pitt-Hopkins syndrome; RTT-like, Rett-like syndrome; SCZ, Schizophrenia; TCF4, transcription factor 4; WB, Western Blot.

Subsequently, we evaluated the ability of mutant TCF4-B¯ to bind DNA using EMSA. For this, *in vitro* translated WT and mutant TCF4-B¯ proteins were incubated with μE5 (CACCTG) containing oligonucleotides and separated on a gel. *In vitro* translated TCF4-B¯ mutants were first visualized by Western blot analysis (Fig. 4*A*) to confirm their translation. The binding of TCF4-B¯ to the μE5 oligonucleotides was specific (Figs. 4, *B*–*D* and S1), as also shown before (5).

First, we studied the effects of disease-related mutations on the ability of TCF4-B¯ homodimers to bind DNA (Figs. 4*B* and S1). The substitutions associated with SCZ and MMID did not change the ability of TCF4-B¯ homodimers to bind DNA (Figs. 4*B* and S1). Of the PTHS-associated mutants, R569W showed lower DNA-binding activity than WT TCF4-B¯, whereas the mutants N585D and A587P completely abrogated the binding of TCF4-B¯ homodimers to the μE5 E-boxes (Figs. 4*B* and S1). RTT-like syndrome associated mutant S253R homodimers displayed slightly reduced DNA binding (Figs. 4*B* and S1).

Next, we studied DNA binding activity in the context of intra-TCF4 heterodimers where one of the dimerization subunits contains a WT bHLH domain (Figs. 4*C* and S1). We cotranslated WT or mutant TCF4-B¯ together with the previously described WT bHLH containing VP16-bHLH protein (5) and assayed the binding of the formed dimers to the μE5 E-boxes. No differences from WT TCF4-B¯ were seen in DNA-binding activity of intra-TCF4 heterodimers for mutants associated with SCZ, MMID, or RTT-like syndrome (Figs. 4*C* and S1). In case of the PTHS mutants, the DNA-binding ability of mutant N585D was partially rescued in the context of TCF4 intra-heterodimers compared with the mutant homodimers, whereas the mutants R569W and A587P displayed no change in DNA-binding activity in response to dimerization with a WT bHLH subunit, showing very low or no DNA binding, respectively (Figs. 4*C* and S1).

Finally, we studied the effect of the disease-associated mutations on the DNA-binding ability of TCF4-B¯ heterodimers with its interaction partner ASCL1. When translated alone, ASCL1 was not able to bind to the μE5 E-box sequence, but when cotranslated with TCF4-B¯, we detected faster moving protein complexes than TCF4-B¯ homodimers (Figs. 4*D* and S1). All TCF4-B¯ mutants were able to dimerize with ASCL1 and bind DNA as heterodimers (Figs. 4*D* and S1). Notably, dimerization of the PTHS mutants R569W and N585D with ASCL1 alleviated the negative effect of these mutations on the DNA-binding ability of TCF4-B¯, and some DNA binding activity was seen for ASCL1 heterodimers with the A587P mutant as well (Figs. 4*D* and S1).

To summarize, our results show that the RTT-like syndrome and PTHS-associated mutations studied here reduce TCF4 DNA-binding activity to a varying extent. The binding ability is least affected in the case of S253R, more in the case of R569W and N585D, and the most severe effects were seen for mutant A587P. The other studied mutations did not affect DNA binding of TCF4 *in vitro*.

### PTHS- and RTT-like syndrome-associated mutations in TCF4 modulate its ability to initiate transcription in HEK293 cells

We assessed the ability of mutant TCF4 proteins to initiate reporter gene transcription using a previously described luciferase reporter assay system (5). Briefly, HEK293 cells were transfected with vectors encoding for WT or mutant TCF4-B¯, firefly luciferase construct with 12 μE5 E-boxes (CACCTG) in front of a minimal promoter and *Renilla* luciferase construct with phosphoglycerine kinase (PGK) promoter for normalization.

**Figure 5:**
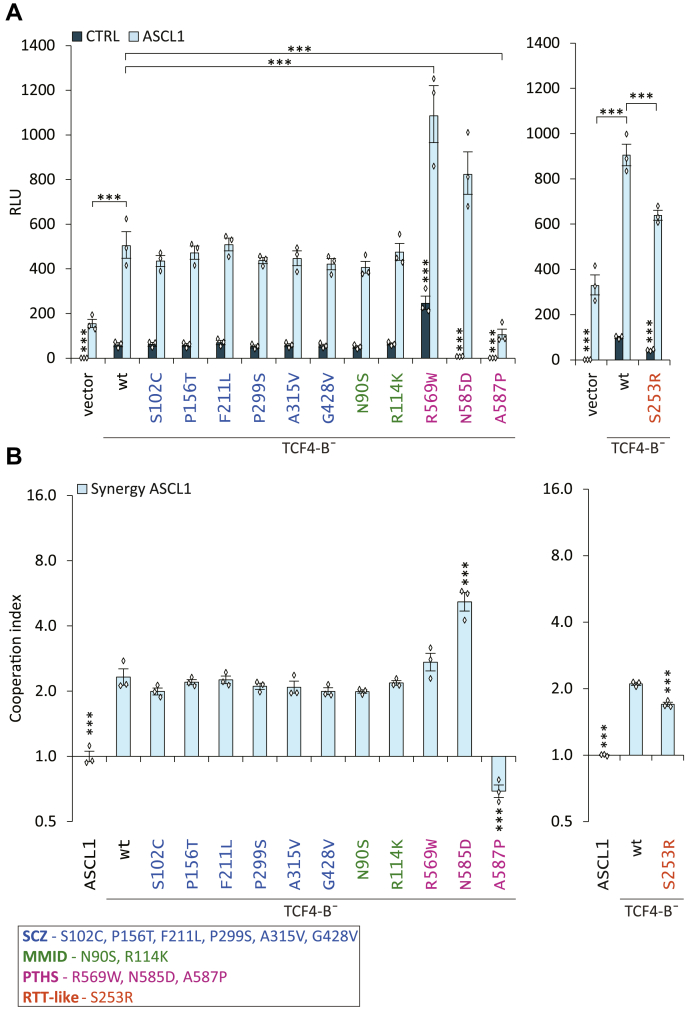
**Missense mutations associated with PTHS alter the ability of TCF4 to activate transcription in HEK293 cells.***A*, luciferase reporter assay with WT or mutant TCF4-B¯. The cells were cotransfected with WT or mutant TCF4-B¯ vectors alone or together with ASCL1, firefly luciferase reporter construct carrying 12 μE5 E-box regulatory sequences (CACCTG) in front of the minimal promoter and *Renilla* luciferase construct with PGK promoter for normalization. *B*, index of cooperation between TCF4-B¯ (WT or mutant) and ASCL1 calculated from data in (*A*). Four (SCZ-associated variations) or three (MMID-, PTHS, and RTT-like syndrome-associated mutations) independent experiments were performed in duplicates. The luciferase data is presented as fold-induced levels above the signals measured from empty vector-transfected (vector) untreated cells. The error bars indicate SEM. For statistical analysis, one-way ANOVA (SCZ, MMID, and PTHS mutants F (25, 50) = 258.2 *p*, < 0.0001; RTT-like syndrome mutant F (1.529, 4.586) = 821.5 *p*, < 0.0001) followed by Holm–Sidak's multiple comparisons test (*A*) or one-way ANOVA (SCZ, MMID, and PTHS mutants F (12, 24) = 59.52 *p*, < 0.0001; RTT-like syndrome mutant F (5, 10) = 1166 *p*, < 0.0001) followed by Dunnett's multiple comparisons test (*B*) was used. The individual data points are shown as *white diamonds*. Statistical significance is shown with *asterisks* and is relative to the cells overexpressing WT TCF4-B¯ or between the bars connected with lines; ∗∗∗*p*, < 0.001. MMID, Mild-to-moderate intellectual disability; PTHS, Pitt-Hopkins syndrome; RLU, relative luciferase units; RTT-like, Rett-like syndrome; SCZ, Schizophrenia; TCF4, transcription factor 4.

SCZ- and MMID-associated variations had no effect on the transactivation by TCF4-B¯ (Fig. 5*A*). Unexpectedly, a 4.1-fold increase (*p* < 0.0001, n = 3) in transcription activation was detected for PTHS-associated mutant R569W. The two other PTHS mutants N585D and A587P showed drastically decreased reporter activity, by 8.2-fold and 118-fold, respectively, (*p* < 0.0001, *p* < 0.0001, n = 3) (Fig. 5*A*). The RTT-like syndrome mutant S253R showed a 2.4-fold (*p* < 0.0001, n = 3) reduction in transactivation (Fig. 5*B*).

All the studied TCF4 missense mutants bound DNA *in vitro* when heterodimerized with ASCL1 according to our EMSA results. Therefore, we next studied whether TCF4-B¯ mutants are functional in driving reporter gene expression when coexpressed with ASCL1 in HEK293 cells. Indeed, coexpression of ASCL1 and TCF4-B¯ increased the transcriptional activity of both the WT and mutant TCF4-B¯ proteins. The SCZ- and MMID-associated mutants showed no aberrations in transcription activation when coexpressed with ASCL1 (Fig. 5*B*). The heterodimers of mutants S253R and A587P displayed a 1.4-fold (*p*< 0.0001, n = 3) and 4.8-fold (*p* < 0.0001, n = 3) reduction in luciferase signals, respectively (Fig. 5*A*). The mutants R569W and N585D increased the transactivation of TCF4-B¯ heterodimers with ASCL1 by 2.2- and 1.6-fold (*p* = 0.0005, 0.0806, n = 3), respectively (Fig. 5*A*).

To assess the synergistic effects between TCF4 and ASCL1, we calculated cooperation indexes based on the data in Figure 5*A* for each WT and mutant TCF4-B¯ protein. The SCZ and MMID mutants and the PTHS-mutant R569W had similar synergistic effects with ASCL1 as WT TCF4-B¯ (Fig. 5*B*). The mutant N585D displayed an increase, whereas mutant S253R showed a slight decrease in the cooperation index compared with the WT TCF4-B¯. A587P largely differed from all the other studied mutants as it displayed an antagonistic effect on the dimerization of TCF4 with ASCL1 (Fig. 5*B*).

Taken together, these results show that the studied disease-related mutations outside the bHLH domain (SCZ and MMID) have no effect on the transactivation of TCF4-B¯, except for the RTT-like syndrome associated mutation S253R. However, mutations within the bHLH region can both increase and reduce the transactivation capability of TCF4-B¯ in HEK293 cells.

### Pitt-Hopkins syndrome-associated missense mutations reduce the transactivation ability of TCF4 in primary cortical neurons

TCF4 is an activity regulated transcription factor in neurons (5, 36). To study whether the disease-associated substitutions in TCF4-B¯ cause aberrations in transcription activation in the cultured primary neurons, we transfected rat cortical and hippocampal primary neurons with plasmids encoding for WT or mutant TCF4-B¯ or TCF4-A¯, ASCL1, firefly luciferase construct carrying 12 μE5 E-boxes (CACCTG) in front of thymidine kinase (TK) promoter and *Renilla* luciferase construct with PGK promoter for normalization. To study the effect of depolarization on transcription activation, the neurons were treated with 25 mM KCl for 8 h or left untreated.

We have previously shown that two of the SCZ-linked missense variations (P299S and G428V) have a slight effect on the ability of TCF4-B¯ to activate transcription in primary neurons. P299S increased reporter activity in basal conditions and G428V in both basal and depolarized conditions (36). Here, we performed a meta-analysis of our previous data (n = 5) and additional experiments carried out in this study (n = 3), confirming our results on the variants P299S and G428V, and in addition revealing a mild increase in the transcriptional activity of A315V variant compared with the WT TCF4-B¯ in basal conditions (Fig. S2*A*).

**Figure 6:**
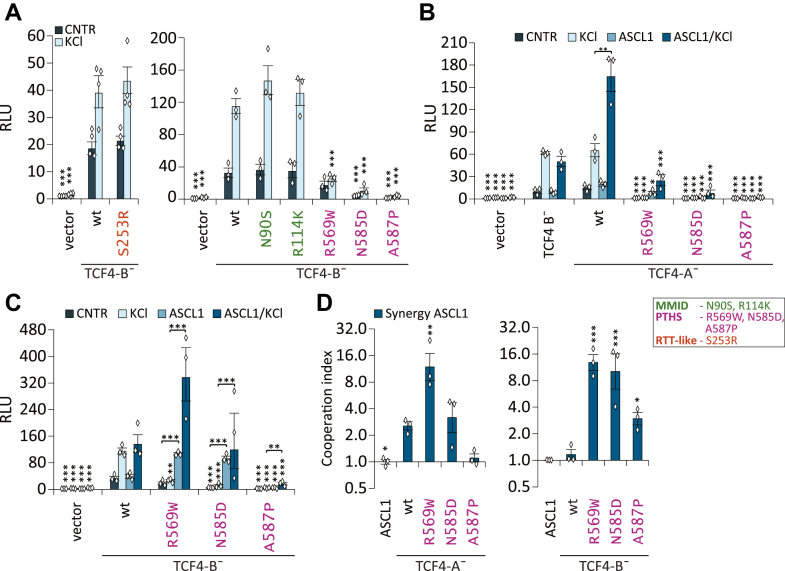
**PTHS-associated missense mutations alter the transcriptional activity of TCF4 in rat cortical and hippocampal primary neurons.***A*–*C*, luciferase reporter assay with WT and mutant TCF4 in rat cortical and hippocampal primary neurons. The cells were cotransfected with WT or mutant TCF4 vectors alone or with ASCL1 vector, firefly luciferase reporter construct carrying 12 μE5 E-box regulatory sequences (CACCTG) in front of TK promoter and *Renilla* luciferase construct with PGK promoter for normalization. The transfected neurons were left untreated (CNTR) or treated with 25 mM KCl for 8 h (KCl) to induce membrane depolarization. Luciferase assays were performed with TCF4-B¯ carrying RTT-like syndrome, MMID, or PTHS-associated mutations (*A*), PTHS mutations containing TCF4-A¯ (*B*) or TCF4-B¯ (*C*). *D*, index of cooperation between ASCL1 and WT or mutant TCF4 calculated from data in (*B*) or (*C*). Three independent experiments were performed in duplicates. The luciferase data is presented as fold-induced levels above the signals measured from empty vector-transfected (vector) untreated cells. The error bars indicate SEM. For statistical analysis, one-way ANOVA (*A*: RTT-like syndrome mutant F (5, 10) = 88.62 *p* < 0.0001; MMID and SCZ mutants F (13, 26) = 70.52 *p* < 0.0001) (*B*: F (27, 54) = 79.12 *p* < 0.0001) (*C*: F (19, 38) = 48.36 *p* < 0.0001) followed by Holm–Sidak's multiple comparisons test (*A*–*C*) or one-way ANOVA (TCF4-A¯ F (5, 10) = 22.05 *p* < 0.0001; TCF4-B¯ F (6, 12) = 31.49 *p* < 0.0001) Dunnett's multiple comparisons test (*D*) was used. The individual data points are shown as *white diamonds*. Statistical significance shown with *asterisks* is relative to cells overexpressing WT TCF4-B¯ (*A* and *C*), WT TCF4-A¯ (*B* and *C*) or between the bars connected with lines; ∗*p*, < 0.05; ∗∗*p*, < 0.01; ∗∗∗*p*, < 0.001. MMID, Mild-to-moderate intellectual disability; PTHS, Pitt-Hopkins syndrome; RLU, relative luciferase units; RTT-like, Rett-like syndrome; SCZ, Schizophrenia; TCF4, transcription factor 4.

The RTT-like syndrome and MMID-associated TCF4-B¯ mutants had similar transactivation ability as WT TCF4-B¯ in both basal and depolarized neurons (Fig. 6*A*). Significant changes in the transcription activation were observed for PTHS mutants (Fig. 6*A*). In basal conditions, the mutants R569W, N585D and A587P displayed decreased luciferase signals by 1.9-fold, 7.3-fold, and 20-fold, respectively, when compared with the WT TCF4-B¯ (*p* = 0.276, *p* < 0.0001, *p* < 0.0001, n = 3) (Fig. 6*A*). In depolarized conditions, the transcriptional activity of all the studied PTHS mutants remained lower than WT TCF4-B¯ in the same conditions (Fig. 6*A*).

Next, we asked whether the disease-associated mutations in TCF4 could affect the cooperation of TCF4 with ASCL1 in neurons, as we found to be the case in HEK293 cells. For this, we first studied the effect of ACSL1 coexpression on the transcriptional activity of WT isoforms TCF4-B¯ and TCF4-A¯ in neurons. We detected no cooperation between ASCL1 and WT TCF4-B¯ in neurons as the presence of ASCL1 did not change the transcriptional activity of TCF4-B¯ in the conditions studied (Fig. 6, *B* and *C*). Contrary to the WT TCF4-B¯, the transcriptional activity of WT TCF4-A¯ was increased 2.5-fold in depolarized neurons when ASCL1 was coexpressed (*p* = 0.0196, n = 3) (Fig. 6*B*), indicating synergistic interaction between the short TCF4 isoform and ASCL1 in neurons (Fig. 6*C*). We detected no cooperation between ASCL1 and WT TCF4-B¯ in neurons as the presence of ASCL1 did not change the transcriptional activity of TCF4-B¯ in the conditions studied (Fig. 6, *B* and *C*).

Based on these results, we decided to study the cooperation of ASCL1 and PTHS mutant proteins in the context of TCF4-A¯ isoform. When PTHS mutant TCF4-A¯ proteins were overexpressed alone in neurons, they showed almost no transcriptional activity in basal or depolarized conditions (Fig. 6*B*). The coexpression of ASCL1 with PTHS mutants resulted in a slight increase of reporter activity, which remained lower from the activity of the WT TCF4-A¯ protein (Fig. 6*B*).

We hypothesised that similarly to TCF4-A¯ PTHS mutants, TCF4-B¯ PTHS mutants may also activate transcription differently from the WT protein when coexpressed with ASCL1. Indeed, we observed that in basal conditions, when the TCF4-B¯ mutants R569W and N585D were coexpressed with ASCL1, the reporter signals were increased 2.8- and 2.4-fold, respectively (*p* < 0.0001, *p* < 0.0001, n = 3), when compared with the WT TCF4-B¯. ASCL1 heterodimers with TCF4-B¯ mutant A587P displayed almost no reporter activity in basal conditions (Fig. 6*D*). In depolarized neurons, the transcriptional activity of ASCL1 heterodimers with TCF4-B¯ was higher in case of R569W, lower in case of A587P, and kept at a similar level to WT in the case of N585D mutation (Fig. 6*D*). For mutations associated with SCZ, MMID, and RTT-like syndrome, no significant changes in transactivation capability of TCF4-B¯ when coexpressed with ASCL1 were detected in any of the studied conditions (n = 3; Fig. S2, *B* and *C*).

The calculated cooperation indexes indicated that R569W mutation increases and A587P abolishes the synergistic effect of ASCL1 with TCF4-A¯ isoform in depolarized neurons (Fig. 6*D*). Differentially from the WT TCF4-B¯ isoform, all studied TCF4-B¯ PTHS mutants cooperate with ASCL1 in neurons (Fig. 6*D*).

Collectively, these results show that the PTHS-associated mutations modify the transcriptional activity of TCF4 in an isoform- and dimerisation partner-dependent manner. The TCF4-A¯ PTHS mutants displayed more severe deficiencies in transcription activation compared with the TCF4-B¯ PTHS mutants. The coexpression with ASCL1 caused little changes in transcription activation of the TCF4-A¯ PTHS mutants, whereas the effect on TCF4-B¯ PTHS mutants was notable. Interestingly, A587P was the only mutation that caused severely reduced reporter signals in all the studied conditions.

## Discussion

**Figure 7:**
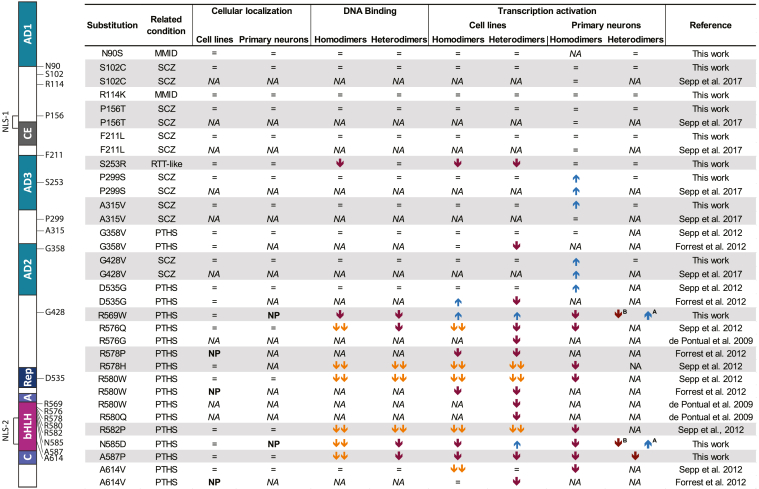
**Summary of the effects of amino acid substitutions on the functionality of TCF4.** The effects of TCF4 amino acid substitutions on cellular localization, DNA binding, and transcription activation alone or together with ASCL1 are shown. *Upwards* and *downwards arrows* denote an increase or decrease in protein function, and "=" denotes no change. Two *downwards arrows* mark dominant negative effects. A or B next to arrows indicate a change specific for TCF4-A¯ or TCF4-B¯. The positions of amino acids affected by disease-related missense mutations and the variations in TCF4-B¯ are shown on the *left*. For more details, see the legend of Figure 1*A*. MMID, Mild-to-moderate intellectual disability; NA, not analysed; NP, nuclear punctae; PTHS, Pitt-Hopkins syndrome; RTT-like, Rett-like syndrome; SCZ, Schizophrenia; TCF4, transcription factor 4.

Previous studies have shown that PTHS-causing missense mutations within *TCF4* alter the function of the protein by regulating transcription activation, dimerization, intracellular localization, and DNA binding (4, 5, 45, 56). In the present study, we analysed 12 novel missense variations and mutations associated with different diseases which are located within and outside the bHLH domain of TCF4. The functional characterization of the missense mutations in TCF4 from this and previous studies is summarized in Figure 7. Altogether, the data reveal that disease-associated mutations in TCF4 mainly modulate DNA binding and transactivation of the protein, whereas transport to the cell nucleus is not affected. The most severe effects are seen for PTHS-associated mutations located in the bHLH region of TCF4. Other disease-related mutations in TCF4 have little or no effect on the functionality of TCF4 in the studied conditions (Fig. 7).

TCF4 has been claimed to be one of the master regulators of SCZ, but the exact mechanisms of how changes in *TCF4* contribute to the development of the disease remain unknown (18, 58). We have previously shown that two (P299S and G428V) out of the six *TCF4* missense variations found in SCZ patients (20, 21) increase the transcriptional activity of TCF4-B¯ in primary cultured neurons (36). Here, we confirm these effects and further show a mild increase in the transcriptional activity of A315V variant. We in addition investigated whether the SCZ-associated missense variations in TCF4-B¯ alter any other functions of TCF4 such as intracellular location, formation of active heterodimers, and binding to DNA but detected no changes. This can partly be explained by the fact that the studied variations were not in any known functionally important regions of the TCF4 protein (except for P156T). We hypothesize that even slight changes in TCF4 functions could be part of a complex network of changes, which contribute to the development of this polygenic disorder (17, 59). Alternatively, the effects of SCZ-associated missense variations in *TCF4* could be more pronounced in conditions, cell types, and/or developmental stages not studied here.

Deletions in the 5′ coding region of the *TCF4* gene have been described in patients with mild nonsyndromic intellectual disability (8, 12). These mutations lead to reduced dosage of the long-TCF4 isoforms. The mild phenotype of MMID compared with PTHS may be explained by the fact that deletions in the 5′ region of the *TCF4* gene do not affect all *TCF4* transcripts, as *TCF4* is transcribed using many alternative 5′ exons located throughout the gene (34). Here, we studied two MMID-associated missense mutations in TCF4 (N90S and R114K), but neither of these had an effect on the functionality of TCF4-B¯, even though N90S is located in AD1. It may be that mutations in the 5′ coding region of *TCF4* cause context-specific effects yet to be discovered and/or are not the only factors underlying the development of MMID in the patients carrying these mutations.

A single mutation (S253R) in the *TCF4* gene has been described in a male patient with RTT-like syndrome. The patient exhibited severe intellectual disability and facial dysmorphisms similar to the phenotype of PTHS (7, 14). In our reporter experiments, mutation S253R was the only TCF4-B¯ mutation outside of the bHLH region that caused significantly reduced reporter activation in HEK293 cells. In addition, EMSA revealed reduced DNA binding of S253R TCF4-B¯. S253R is located in AD3 of the TCF4 in a position that interacts directly with the primary core-promoter recognition factor transcription factor TFIID complex (35). Broadly, TFIID can modulate transcriptional activity of RNA polymerase II, but more specifically, it may also stabilize E-proteins through direct interaction, which may be necessary for the binding of coactivators or repressors to TCF4 (35, 60). It is possible that transcription initiation mediated by the mutant S253R is decreased because of reduced DNA binding or impaired binding of coactivators or increased binding of repressors. This however seems to be a cell-type specific effect as we saw no changes in the transcriptional activity of S253R TCF4-B¯ in cultured neurons.

We confirm that missense mutations in the bHLH region of TCF4 have severe effects on the functioning of the protein, especially mutations affecting the arginine residues. PTHS-associated mutants R569W and N585D in TCF4-B¯ displayed aberrant intranuclear localization and were detected as dots in primary neuron cultures. Previous experiments from our workgroup (5) suggest that the protein aggregates observed as nuclear dots could refer to protein destabilization and misfolding. Therefore, it is possible that the mutations R569W and N585D affect the folding stability of TCF4. Another explanation for the development of intranuclear puncta may be related to the localization of R569W and N585D in the recently described NLS-2 (41). R569W and N585D could cause NLS-2 to dysfunction leaving NLS-1 the only functional NLS. This suggests that NLS-1 is necessary for transport of TCF4 to the nucleus, whereas NLS-2 may be involved in intranuclear localization as NLS-2 present in TCF4-A is not sufficient to cause strict nuclear localization as seen for TCF4-B. However, the detected aberrant localization of R569W and N585D in the nucleus is cell type-dependent because we detected changes in the localization of these mutants in neurons, and not in HEK293 cells. Whether the localization of TCF4-B¯ mutants as puncta may be due to the lack of a functional NLS-2 remains to be clarified.

The reporter experiments revealed that R569W increased the transcriptional activity of TCF4-B¯ in HEK293 cells, whereas in neurons, the transcriptional activity of R569W TCF4-B¯ was reduced. Interestingly, the coexpression of R569W TCF4-B¯ with ASCL1 resulted in the increased activity relative to WT TCF4-B¯. This observation is supported by experiments by Forrest *et al*. who showed that PTHS-associated mutations present variable effects depending on the context (56). The detected cell-type specific effects on transcription activation could be due to regulatory partners that are present in HEK293 cells but are absent in rat primary neuron cultures or vice versa. Similar experiments in different cell types, including neurons, derived from human iPSCs would be instrumental in elucidating the cell-type specific activity of TCF4 in the human context.

N585D TCF4-B¯ displayed impaired transcription activation, whereas its heterodimers with ASCL1 were transcriptionally more active than those of the WT TCF4-B¯. This implicates that N585D disrupts DNA binding of homodimers, whereas it enhances the formation of heterodimers or heterodimer binding of TCF4-B¯. This could be caused by structural changes caused by the mutation as N585 forms hydrogen bonds with DNA backbone in TCF4 homodimers. N585D could in part disrupt the α-helical structure important for homodimer DNA-binding, and the other bHLH dimerization partners rescue the damaging effects. This is also in accordance with our EMSA results because we saw that the dimerization of N585D with ASCL1 alleviates the negative effect of the mutation on DNA binding to some extent.

We have previously shown that a mutation outside of the basic DNA-binding region but within the bHLH domain (A614V) almost completely abrogates DNA binding and transcription activation capability of TCF4. However, the negative effect of that mutation was rescued by dimerization with ASCL1 (5). Here, we show that mutation A587P, which is in the first helix of the bHLH region, completely abrogates the transcriptional activity of TCF4-B¯ and TCF4-A¯. The negative effect of A587P on transcription activation was only partly rescued by interaction with ASCL1 in HEK293 cells, but not in primary neuron cultures. Our analysis shows that mutation A587P does not interfere with nuclear localization but rather impairs DNA binding of mutant A587P dimers. A587 packs against residue L611 in helix 2 of the opposite monomer, therefore, the mutation in this region can impair the formation of functionally active dimers. Combined, our results indicate that A587P may act as a dominant-negative mutation, which results in the formation of inactive dimers that cannot bind to DNA.

Different TCF4 isoforms present varying transactivation capabilities between cell types (34, 36). Here, we show that amino acid substitutions in TCF4 lead to variable effects depending on the protein isoform. Contrary to the respective TCF4-B¯ mutants, R569W and N585D TCF4-A¯ mutants displayed drastically lower transcription activation in cultured primary neurons than the WT protein. This may arise from the differences in the presence of protein domains. TCF4-B¯ carries functional protein regions AD1, CE, and NLS-1, which are not present in the short isoform TCF4-A¯. It would be of interest to elucidate whether deleting one or more of these functional regions in TCF4-B¯ mutant proteins results in similar effects on transcription activation as seen for TCF4-A¯ mutants.

Our results indicate that the heterodimerization of TCF4 mutants may both decrease and increase the transcriptional activity of TCF4. As TCF4 dimerization partners are differentially expressed during development of the central nervous system (27), it is possible that the dysregulation of TCF4’s activity in response to the studied missense mutations is much more complex and cannot be modelled in cell cultures. There may exist a regulatory mechanism that defines which dimerization partners can interact with TCF4 during development that is impaired in response to missense mutations in TCF4. As TCF4 expression levels in the cerebral cortex of rodents and humans are highest around birth (42, 61) it would be interesting to study the effects of TCF4 missense mutations on the functionality of TCF4 *in vivo* at perinatal stages.

The effects of SCZ, MMID, and RTT-like syndrome associated mutations and variations on the functioning of TCF4 were only studied in the context of TCF4-B¯ which may be the reason why we saw mild or no effects of SCZ, MMID, and RTT-like syndrome associated mutations and variations on the functioning of TCF4. However, 18 N-terminally distinct protein isoforms with different transcriptional activities are encoded by the human *TCF4* gene, of which the most studied long-TCF4 isoform is isoform TCF4-B¯ (34, 36). The mutations N90S, R114K and P156T are present in the major TCF4 protein isoforms TCF4-B, -C, -E, -F, -D, and -G, expressed in the central nervous system (34). All the other studied mutations and variations are in the C-terminal end of TCF4 and are present in all the TCF4 protein isoforms. Because our experiments focused on the effect of mutations and variations on TCF4-B¯ and TCF4-A¯, it remains unknown whether the studied aberrations could present varying effects depending on different TCF4 protein isoforms. In addition, Sepp *et al*. (5, 36) and Forrest *et al*. (56) have studied the effects of TCF4 missense mutations on DNA binding and transcription activation using the CACCTG E-box sequence. A ChIP-seq study in SH-SY5Y cells has shown that TCF4-A and TCF4-B display enrichment for the CATCTG E-box sequence (62). Still, the binding specificity of TCF4 to various E-box combinations is regulated by dimerization partners. For example, the preferred E-box motif of ASCL1 is CAGCTG (63). It remains to be elucidated whether changes in E-box sequences can affect the activity of TCF4. Our experiments were carried out using cell cultures, but the use of different model systems such as differentiated stem cells or organoids in addition to *in vivo* studies could provide more insight on how different mutations affect TCF4 and whether the E-box binding specificity is affected by amino acid substitutions in TCF4.

To conclude, the results of this and previous studies show that missense mutations in TCF4 affect transcription activation and DNA binding of the protein, which can be partially rescued by the formation of heterodimers with dimerization partners such as ASCL1. Further studies are needed to understand how or whether SCZ and MMID associated missense variations and mutations in TCF4 may contribute to disease pathogenesis. The most severe effects on protein function, including complete loss-of-function, were seen for PTHS-associated mutations in the bHLH domain of TCF4. Together with previous work, our study gives an overview of the functional consequences of disease-related missense mutations in *TCF4* providing a basis for the interpretation of *TCF4* missense variants and their potential pathogenicity.

## Experimental procedures

### Constructs

Schizophrenia-associated missense variations (S102C, P156T, F211L, P299S, A315V, and G428V), RTT-like syndrome associated missense mutation (S253R), mild-to-moderate intellectual disability associated missense mutations (N90S and R114K) and Pitt-Hopkins syndrome associated missense mutations (R569W, N585D, and A587P) were introduced into pcDNA3.1/TCF4-B¯ vectors (34) *via* site-directed mutagenesis with complementary oligonucleotides (Table S1). For all constructs, the initial CMV promoter was substituted with elongation factor 1 α promoter, as described before (36). All the mutations were mapped and numbered according to the TCF4-B^+^ NCBI reference sequence NP_001077431.1.

For pcDNA3.1/EF1a/E2-TCF4-B¯ vectors, E2-tag was cloned from pQM/CMV/E2_N (Icosagen) and inserted in front of *TCF4* coding sequence. pcDNA.3.1/EF1a/TCF4-A¯ has been described (36). pcDNA3.1/EF1a/TCF4-A¯ vectors with missense mutations G428V, R569W, N585D, and A587P were generated from corresponding pcDNA3.1/EF1a/TCF4-B¯ vectors by replacing the fragment between the KpnI and MluI restriction sites (Thermo Scientific). All the vectors were verified by sequencing.

pcDNA3.1/ASCL1, pcDNA3.1/NEUROD2-E2, pACT-bHLH, and luciferase reporter assay constructs pGL4.29[luc2P/12μE5/min/Hygro] with 12 E-boxes in front of minimal promoter, pGL4.29[luc2P/12μE5/TK/Hygro] with 12 E-boxes in front of thymidine kinase promoter, and pGL4.83[hRlucP/PGK1/Puro] with phosphoglycerate kinase 1 promoter have been previously described (5, 34, 36).

### Protein structure visualization

TCF4 bHLH model was previously generated using E-protein E47 (TCF3 isoform) crystal structure coordinates (5). Protein Data Bank compatible files and PyMOL Molecular Graphics System session files were used for the visualization of the protein structure. The analysis of protein-DNA and protein-protein interactions was made in PyMOL Molecular Graphics System (version 1.7.4.5 trial for educational use). The ribbon drawing of TCF4-B^+^ (NP_001077431.1) bHLH homodimer structure was generated using YASARA View (version 17.4.17, YASARA Biosciences GmbH) (64).

### Cell culture and transfection

Human embryonic kidney HEK293 cells were grown in Minimal Essential Medium with Earle’s Salts (PAA Laboratories) supplemented with 10% Fetal Bovine Serum (SeraPlus and PAN Biotech), 100 U/ml penicillin, and 0.1 mg/ml streptomycin (Gibco) at 37 °C in 5% CO_2_. Rat primary cortical and hippocampal mixed neuronal cultures were plated from embryonic day 20 to 21 Sprague-Dawley rat fetuses and maintained, as described previously (65). The protocols involving animals were approved by the ethics committee of animal experiments at the Ministry of Agriculture of Estonia (Permit Number: 45). All the experiments were performed in accordance with the relevant guidelines and regulations.

The HEK293 cells were transfected using polyethylenimine (Sigma-Aldrich) with polyethylenimine to DNA ratio 2:1. For Western blot analysis, the cells grown on 6-well plates were transfected with 1.8 μg of TCF4-B¯ encoding vector and with 0.2 μg of pEGFP vector to evaluate transfection efficiency. For luciferase reporter assays, the cells grown on 48-well plates were transfected with 0.1875 μg of effector protein construct(s) (TCF4-B¯ alone or TCF4-B¯ and ASCL1 together), 0.1875 μg of firefly luciferase construct pGL4.29[luc2P/12μE5/min/Hygro], and 0.01 μg of *Renilla* luciferase construct pGL4.83[hRlucP/PGK1/Puro]. In case of cotransfection of effector protein constructs, pcDNA3.1/EF1a/TCF4-B¯ and pcDNA3.1/ASCL1 were added in a ratio of 2:1. For nuclear redirection assay, 0.2 μg of pcDNA3.1/EF1a/TCF4-A¯ and 0.1 μg pcDNA3.1/ASCL1 or pcDNA3.1/NEUROD2-E2 were transfected to the HEK293 cells grown on 48-well plates.

The neurons plated on 48-well plates were transfected on 6 days *in vitro* using Lipofectamine 2000 (Invitrogen) with a reagent to DNA ratio 2:1. For luciferase reporter assays, 120 ng of effector protein construct(s) (TCF4-B¯ alone or TCF4-B¯ and ASCL1 together), 60 ng of firefly luciferase construct pGL4.29[luc2P/12uE5/TK/Hygro] and 20 ng of *Renilla* luciferase pGL4.83[hRlucP/PGK1/Puro] were used. In case of cotransfection of effector protein constructs, pcDNA3.1/EF1a/TCF4-B¯ and pcDNA3.1/ASCL1 were added in a ratio of 2:1. 2 days posttransfection, and the neuronal cultures were treated with 25 mM KCl for 8 h where indicated. For protein localization assays, 0.2 μg of pcDNA3.1/EF1a/TCF4 (HEK293 cells) or pcDNA3.1/EF1a/E2-TCF4 (neuronal cultures) plasmids were transfected to the cells grown on 48-well plates using Lipofectamine 2000 (Invitrogen), with a reagent to DNA ratio 2:1.

### Cell extracts and Western blotting

The cells were lysed 48 h posttransfection in radioimmunoprecipitation assay buffer (150 mM NaCl, 1% NP-40, 0.5% sodium deoxycholate, 0.2% sodium dodecyl sulfate, 50 mM Tris-HCl, 1× Protease inhibitor cocktail Complete (Roche), and 1 mM DTT). The protein concentrations were measured with Pierce BCA Protein Assay Kit (Thermo Scientific).

Equal amounts of protein were separated in 8 to 10% SDS-polyacrylamide gel and transferred to Immobilon-P polyvinylidene fluoride membrane (Millipore). The membrane was blocked with 5% skimmed milk in PBS-0.1% Tween 20 (PBST) (Sigma-Aldrich) at room temperature and incubated with primary and secondary antibody solutions in 2% skimmed milk in PBST. The antibodies were used in the following dilutions: rabbit polyclonal anti-TCF4 (CeMines) 1:1000, mouse monoclonal anti-E2 (5E11, Icosagen,) 1:5000, mouse monoclonal anti-GAPDH (MAB374, Millipore) 1:4000, horseradish peroxidase-conjugated goat anti-mouse/rabbit IgG (Thermo Scientific) 1:5000. For chemiluminescent reaction, SuperSignal West Femto Maximum Sensitivity Substrate Kit (Thermo Scientific) was used, and the reaction was visualized with ImageQuant LAS4000 camera system (GE Healthcare).

### Immunocytochemistry

For protein localization and nuclear redirection assays, the cells were grown on poly-L-lysine coated cover slips. The cells were fixed 48 h posttransfection in 4% paraformaldehyde (Applichem) in PBS for 15 min, then treated with 50 mM ammonium chloride (Scharlau) in PBS, and permeabilized with 0.5% Triton X-100 (Amresco) solution in PBS. After each reaction, cover slips were washed with PBS. The cells were blocked with 2% bovine serum albumin (BSA, Naxo) in PBS for 1 h at room temperature and incubated first with primary and then with secondary antibodies in 0.2% BSA-PBS solution. The antibodies were diluted as follows: rabbit polyclonal anti-TCF4 (CeMines) 1:200, mouse monoclonal anti-E2 (5E11, Icosagen,) 1:1000, mouse monoclonal anti-MASH1 (ASCL1) (24B72D11.1, BD Pharmingen) 1:200, mouse monoclonal anti-tubulin-β (E7, DSHB) 1:1200, rabbit polyclonal anti-tubulin-β III (T2200, Sigma-Aldrich) 1:400, and Alexa Fluor 488- or Alexa Fluor 568-conjugated F(ab’)2 fragment of goat anti-mouse/rabbit IgG (Invitrogen) 1:2000. 1 μg/ml of Hoechst 33342 was included in secondary antibody solution to visualize nuclei. The cover slips were washed with PBST after both reactions. Finally, the cover slips were washed with water and mounted with Mowiol 4-88 mounting medium (Polysciences, Inc). The whole area of the samples was analysed by confocal microscopy (LSM 510 Duo Zeiss) using lasers Argon/2 (for Alexa Fluor 488, 488 nm), DPSS 561-10 (for Alexa Fluor 568, 568 nm) and Diode 405-50 (for Hoechst 33342, 405 nm), and objective lenses PlanApo 63× oil or PlanApo 100× oil (NA 1.4). Picture panels were made using Imaris (version 6.4.2, Bitplane), Photoshop, and Illustrator (Adobe) software. All the experiments were performed twice.

### *In vitro* translation and EMSA

TnT T7 Quick Coupled Transcription/Translation System (Promega) was used according to manufacturer’s instructions, using unlabeled methionine, to produce *in vitro*-translated proteins. For cotranslations, construct ratio of 2:1 was used in favor for the longer product-pcDNA3.1/EF1a/TCF4-B¯:pcDNA3.1/ASCL1 2:1 and pcDNA3.1/EF1a/TCF4-B¯:pACT-bHLH 2:1. Previously described μE5 and mutated μE5 oligonucleotides (5) were ^32^P labeled with T4-polynucleotide kinase (Thermo Scientific). The sense and antisense oligonucleotides were annealed in annealing buffer (62.5 mM NaCl and 1.25 mM EDTA) in a 95 °C water bath, which was left to cool to room temperature overnight. The binding reaction was done in a 15 μl reaction buffer (20 mm Hepes-KOH, pH 7.9, 50 mM KCl, 5% glycerol, 1 mm EDTA, 1 mm DTT, 13.3 ng/μl poly(dI-dC), and 0.1 mg/ml BSA), which included 1 μl of *in vitro*-translated protein mixture and 0.1 pmol of ^32^P-labeled μE5 oligonucleotides. Specificity of binding to μE5 boxes was tested by adding 1 pmol of unlabeled-μE5 oligonucleotides to the reaction mixture. The DNA-protein complexes were resolved in a 5% nondenaturing polyacrylamide gel containing 0.25× TBE, 0.01% NP-40, and 2.5% glycerol and visualized by autoradiography.

### Luciferase reporter assay

For luciferase reporter assays, the cells were lysed 48 h posttransfection in Passive Lysis Buffer (Promega) and kept at −80 °C overnight. The reporter luminescence signals were obtained in duplicates with Dual-Glo Luciferase assay kit (Promega) according to manufacturer’s protocol and measured with GENios pro (Tecan) plate reader. All the experiments were repeated 3 times or more where indicated. In addition, the data from Sepp *et al*. 2017 (n = 5) (36) and this work (n = 3) was combined for more statistical power (n = 8) to better describe the effects of SCZ-related missense mutations on the functionality of TCF4. For data analysis, the background signals were subtracted, and firefly luciferase signals were normalized to *Renilla* luciferase signals. The data were log-transformed and auto-scaled. GraphPad Prism 7 (version 7.00) was used for one-way or two-way ANOVA statistical analysis followed by Dunnett’s or Holm–Sidak's multiple comparisons test. The data were then back transformed into the original scale for graphical representation. Statistical significance was calculated for comparisons with WT TCF4, unless indicated otherwise. The cooperation index was calculated, as described previously (5, 66). An index value of higher or lower than 1 implies synergism or antagonism, respectively. For statistical analysis, one-way ANOVA followed by Dunnett’s post hoc test was used.

## Data availability

The data that support the findings of this study are available from the corresponding author upon reasonable request.

## Supporting information

This article contains supporting information.

## Conflict of interest

The authors declare that they have no conflict of interest with the contents of the article.
