# Supplementary material for: Functional consequences of TCF4 missense substitutions associated with Pitt-Hopkins syndrome, mild intellectual disability, and schizophrenia
Source: J Biol Chem. 2021 Nov 6;297(6):101381. doi: 10.1016/j.jbc.2021.101381 (PMC8648840; doi:10.1016/j.jbc.2021.101381)
Supplement: Figures S1 and S2 and Table S1 [file mmc1.docx]

**Functional consequences of TCF4 missense substitutions associated with Pitt-Hopkins syndrome, mild intellectual disability, and schizophrenia**

Alex Sirp, Kaisa Roots, Kaja Nurm, Jürgen Tuvikene, Mari Sepp, Tõnis Timmusk

Supporting material included:

- Table S1, p. S-2
- Figure S1, p. S-3
- Figure S2, p. S-4, S-5

**Table S1: Oligonucleotides used for site-directed mutagenesis of SCZ, MMID, RTT-like syndrome and PTHS associated variations and mutations**

| **Specificity** | **Oligonucleotide sequence (5’ – 3’)** |
| --- | --- |
| N90S_F | GGTCACATGACA**G**TCTCTCTCCACCTTTTGTC |
| N90S_R | GACAAAAGGTGGAGAGAGA**C**TGTCATGTGACC |
| S102C_F | CCAGAATACAA**T**GTAAAACAGAAAGGGGC |
| S102C_R | GCCCCTTTCTGTTTTAC**A**TTGTATTCTGG |
| R114K_F | CTCATCTTATGGGA**A**AGAATCAAACTTAC |
| R114K_R | GTAAGTTTGATTCT**T**TCCCATAAGATGAG |
| P156T_F | GTATTCTAGCAATAAT**A**CCCGAAGGAGGCCTC |
| P156T_R | GAGGCCTCCTTCGGG**T**ATTATTGCTAGAATAC |
| F211L_F | GCAACCAGCACTTT**A**CCTAGCTCCTTCTTCATGC |
| F211L_R | GCATGAAGAAGGAGCTAGG**T**AAAGTGCTGGTTGC |
| S253R F | CACAGTCCAGCAG**G**TACTGTAGCCTGCAT |
| S253R R | ATGCAGGCTACAGTA**C**CTGCTGGACTGTG |
| P299S_F | CCTGTACGCCT**T**CTGCCAACGGG |
| P299S_R | CCCGTTGGCAG**A**AGGCGTACAGG |
| A315V_F | GGAAGCGGGGCAG**T**CGGCAGCTCCCAGACTGG |
| A315V_R | CCAGTCTGGGAGCTGCCG**A**CTGCCCCGCTTCC |
| G428V_F | GGACCTTCTCATAATG**T**AGCCATGGGTGG |
| G428V_R | CCACCCATGGCT**A**CATTATGAGAAGGTCC |
| R569W_F | GCGTGAGAAGGAG**T**GGAGGATGGCCAAC |
| R569W_R | GTTGGCCATCCTCC**A**CTCCTTCTCACGC |
| N585D_F | GGTCCGTGACATC**G**ACGAGGCTTTCAAAG |
| N585D_R | CTTTGAAAGCCTCGT**C**GATGTCACGGACC |
| A587P_F | GTGACATCAACGAG**C**CTTTCAAAGAG |
| A587P_R | CTCTTTGAAAG**G**CTCGTTGATGTCAC |


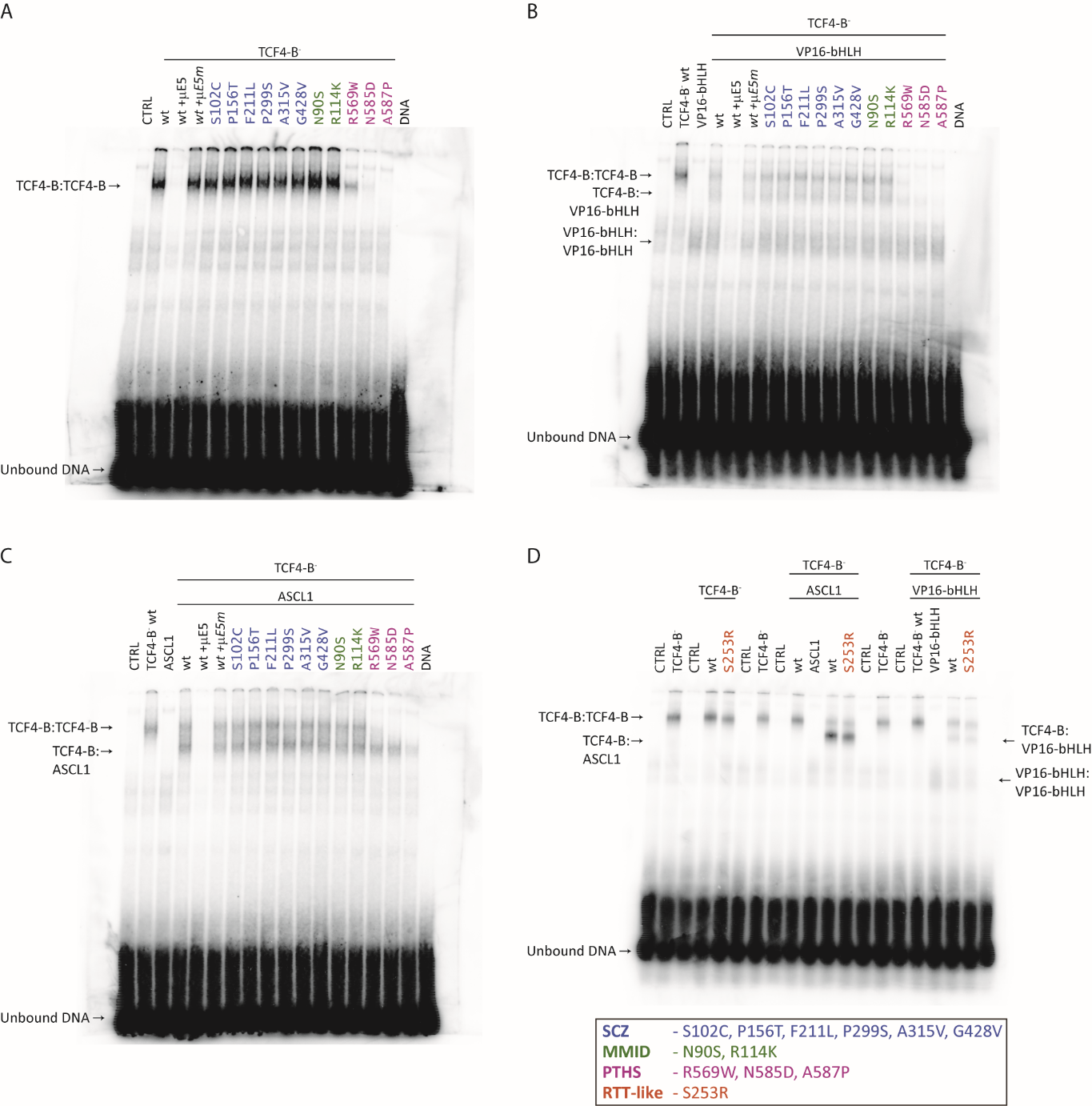


**Figure S1. DNA binding of TCF4 homo- and heterodimers is impaired by mutations associated with PTHS.** Electrophoretic mobility shift assay to study the binding of *in vitro*-translated wt or mutant TCF4-B**¯** proteins to the 32^P^ labelled μE5 E-box (CACCTG) containing oligonucleotide as **(A, D)** homodimers (TCF4-B:TCF4-B), **(B, D)** intra-TCF4 heterodimers consisting of one wt or mutant TCF4-B¯and one wt VP16-bHLH subunit (TCF4-B:VP16-bHLH) and **(C, D)** heterodimers with ASCL1 (TCF4-B:ASCL1). Unlabelled wt (μE5) or mutated (μE5m) E-box oligonucleotides were added to the binding mixture for competition where indicated in italics. kDa, kilodalton.


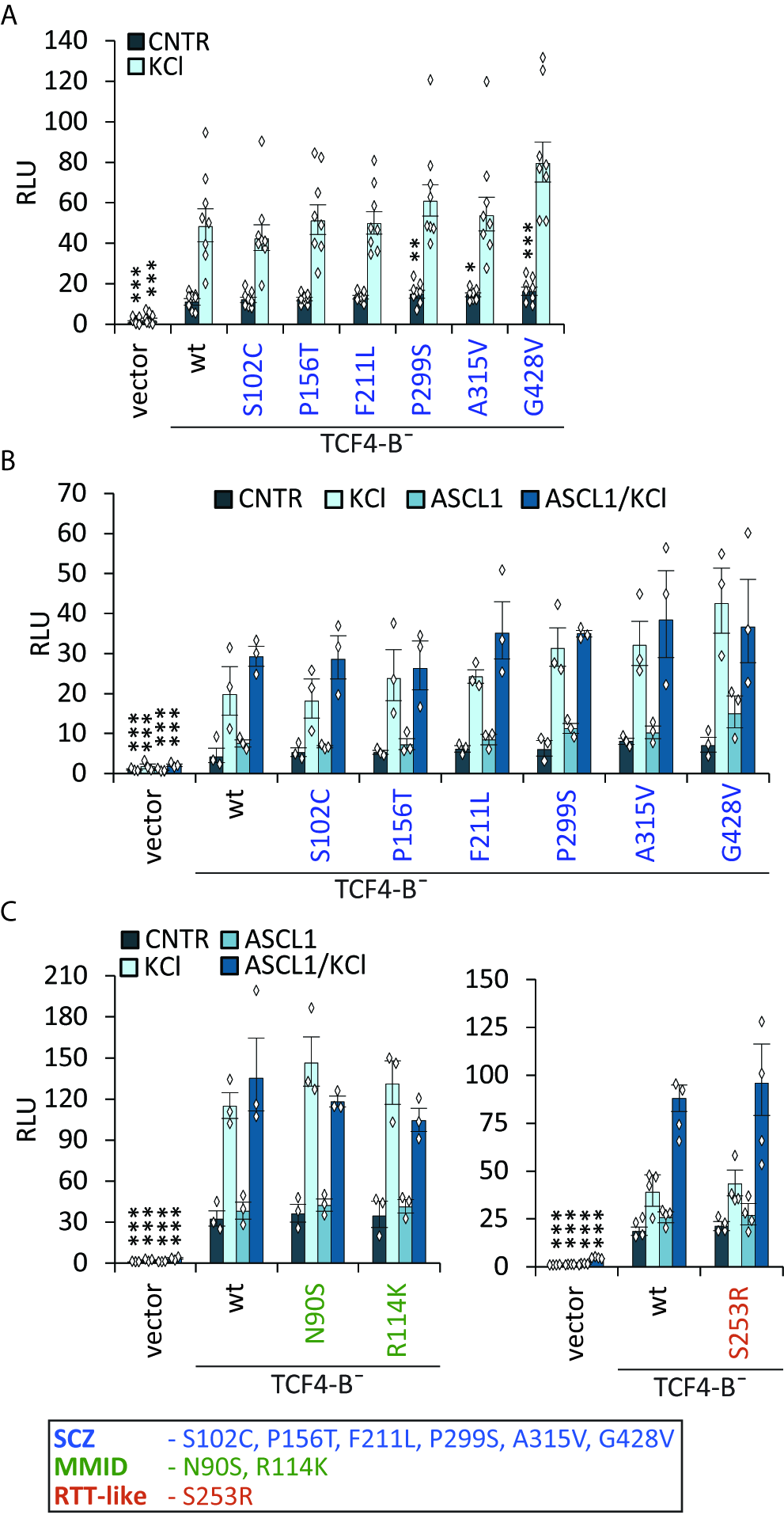


**Figure S2. SCZ related missense variations P299S, A315V and G428V increase the transcriptional activity of TCF4 in rat cortical and hippocampal primary neurons.** Luciferase reporter assay with wt and mutant TCF4 in rat cortical and hippocampal primary neurons. Cells were co-transfected with wt or mutant TCF4 vectors alone (A, B, C) or with ASCL1 vector (B, C), firefly luciferase reporter construct carrying 12 μE5 E-box regulatory sequences (CACCTG) in front of TK promoter and *Renilla* luciferase construct with PGK promoter for normalization. Transfected neurons were left untreated (CNTR) or treated with 25 mM KCl for 8 h (KCl) to induce membrane depolarization. Luciferase assays were performed with TCF4-B¯ carrying SCZ (A, B), MMID or RTT-like syndrome (C) associated mutations. The data is presented as fold induced reporter levels above the signals measured from empty vector-transfected (vector) untreated cells from eight (8) or three (B, C) independent experiments. In A, data from this (n=3) and previous study (57) were integrated. Error bars indicate SEM. For statistical analysis, one-way ANOVA (B: F (17, 34) = 43.45 P<0.0001) (C: MMID mutants F (1.974, 3.948) = 39.46 P=0.0025; RTT-like mutant F (1.89, 5.671) = 116.2 P<0.0001) followed by Holm-Sidak's multiple comparisons test (B, C) or two-way ANOVA (F (1, 7) = 270.4 P<0.0001, F (7, 49) = 12.27 P<0.0001) followed by Dunnet's multiple comparisons test (A) was used. Individual data points are shown as white diamonds. Statistical significance shown with asterisks is relative to cells overexpressing wt TCF4-B¯; *P<0.05; **P<0.01; ***P<0.001. RLU – relative luciferase units.
